# Variation in Atmospheric ^137^Cs and the Carriers in Aerosol Samples Obtained from a Heavily Contaminated Area of Fukushima Prefecture

**DOI:** 10.3390/toxics14010088

**Published:** 2026-01-19

**Authors:** Huihui Li, Peng Tang, Kazuyuki Kita

**Affiliations:** 1 School of Chemical Engineering, Sichuan University of Science and Engineering, Zigong 643000, China; janet.lihuihui@gmail.com; 2 Tianjin Key Laboratory of Brine Chemical Engineering and Resource Eco-Utilization, College of Chemical Engineering and Materials Science, Tianjin University of Science and Technology, No. 29, 13th Street, Binhai New District, Tianjin 300457, China; 3 Graduate School of Science and Engineering, Ibaraki University, 2-1-1 Bunkyo, Mito 310-8512, Ibaraki, Japan; kazuyuki.kita.iu@vc.ibaraki.ac.jp

**Keywords:** atmospheric ^137^Cs, ^137^Cs carriers, resuspension, aerosol samples, Fukushima Daiichi Nuclear Power Plant accident

## Abstract

Even a decade after the Fukushima Daiichi Nuclear Power Plant (FDNPP) accident on 11 March 2011, fluctuations in atmospheric ^137^Cs were still observed, and explanations for the fluctuations and their carriers remained elusive. In this study, small fluctuations within 0.0002 Bq∙m^−3^ were still detected in aerosol samples obtained from January to April, and slightly higher levels of atmospheric ^137^Cs were observed from May to September in a heavily contaminated area of Fukushima prefecture. Specifically, it is demonstrated that the ^137^Cs carriers in the aerosol samples were a combination of carbon-containing particles and aluminum-containing particles (Al particles dominated, with the percentage being 68%) in early May, whereas the main ^137^Cs carriers were carbonaceous particles, with the average percentage being 88% in September and at the end of May, using fluorescent upright microscope and scanning electron microscope equipped with an energy-dispersive X-ray spectrometer quantitatively. Additionally, small particles (less than 2 μm) and medium particles (2–8 μm) of carbonaceous particles had a higher level in the aerosol samples of May and September. Specifically, bacteria (1–1.8 μm) and spores (1.8–10 μm) had a linear relationship with the distribution of atmospheric ^137^Cs in the aerosol samples of September. In addition, temperature and precipitation were the main impact factors affecting the distribution of ^137^Cs and their carriers. This observation further suggests that there is still a need for long-term monitoring of atmospheric ^137^Cs.

## 1. Introduction

### 1.1. The Fukushima Daiichi Nuclear Power Plant Accident

The Fukushima Daiichi Nuclear Power Plant (hereinafter referred to as FDNPP, 37°25′ N, 141°02′ E, located on the Northeastern Pacific Ocean coast of Honshu, about 200 km far from Northeast Tokyo) was one of the nuclear power plants of the Tokyo Electric Power Company (TEPCO). At 14:46 on 11 March 2011, a large-scale earthquake (also known as the Great East Japan Earthquake) with a magnitude of 9.0 occurred in the Tohoku region [1]. At the time of the disaster, Units 1–3 were normally operating, and Units 4–6 were scheduled to be shut down for maintenance [2]. The shaking caused by the earthquake and subsequent tsunami-induced flooding disaster resulted in the loss of the electricity of FDNPP, which was needed to run and cool the reactors and spent-fuel-pools normally [3]. Therefore, a large amount of hydrogen gases was generated by the reaction of uncontrollable residual heat with metal in the units. Despite venting and water-injection operations in Units 1–3, hydrogen explosions were not avoided in Unit 1 and Unit 3. Thus, large amounts of radionuclides were released into the atmosphere and deposited on land and in the Pacific Ocean [4].

### 1.2. Deposition and Distribution of ^137^Cs

A variety of radionuclides were released into the atmosphere and deposited in the terrestrial and marine environments, which could cause health and environmental contamination, such as contamination problems on soil surfaces, in water, in agricultural products and animal by-products, etc. The main radioactive radionuclides were iodine 131 (^131^I), cesium 134 (^134^Cs), cesium 137 (^137^Cs), and xenon 133 (^133^Xe). As shown in Table S1, the estimated amount of radionuclides released from the FDNPP accident were published by the Japan Atomic Energy Agency (JAEA) on 12 April 2011, and NISA (Nippon Individual Savings Account) on 12 May 2011, respectively [5]. ^137^Cs has a longer half-life of approximately 30 years, which has attracted much more attention of researchers than other short half-life radionuclides, for example, ^131^I and ^133^Xe (which has a short half-life of 8 days and 5 days, respectively) [6]. Additionally, the physicochemical properties of ^137^Cs are similar to those of potassium. Therefore, the soluble ^137^Cs was easily absorbed by animals and plants. When ^137^Cs entered the body of animals, it was mainly retained in bone and muscle tissue [7]. Thus, the long-term monitoring and analysis of ^137^Cs is particularly important for understanding the fate of ^137^Cs.

### 1.3. Resuspension of ^137^Cs

It was reported that about 2.7 PBq of ^137^Cs was deposited on the ground, of which 60–67% was deposited in the forests [8]. This deposited ^137^Cs and its carriers can easily become the secondary contamination sources. Specifically, it was demonstrated [9] that the monthly deposition speed of ^137^Cs decreased with an apparent half-life of 11–14 days during the period of March–June 2011. Also, the second peak in monthly deposition of ^137^Cs was observed in February–April 2012, which may be ascribed to the resuspension of ^137^Cs-bearing particles (CsMPs) [10]. In addition, most of the FDNPP-derived ^137^Cs was deposited on the topsoil and remained in the soil surface layer as a potential secondary source of atmospheric ^137^Cs [11]. The resuspension process of ^137^Cs could be defined as the redistribution of the deposited ^137^Cs into the atmosphere by wind or anthropogenic processes [12,13,14]. According to Kajino et al., the respective resuspension rates of ^137^Cs were estimated to be 1 × 10^−6^ day^−1^ and 2 × 10^−6^ day^−1^ for bare soil surfaces and forest ecosystems, revealing a seasonal change in which the high level of ^137^Cs could be observed in warm seasons and the low could be obtained in cold seasons [14]. Specifically, by considering different soil textures, Ishizuka et al. [15] thought that possible carries of ^137^Cs may be suspended soil dust carrying ^137^Cs and modeled the particle size distribution. Kinase et al. [16] and Igarashi et al. [17] discussed possible ^137^Cs carriers and showed that soil particles [18] can be significant in the springtime and that bioaerosols such as pollens [19], spores [17], and microorganisms [16] can be major possible host particles in summer and autumn. Moreover, it was directly demonstrated by Higaki et al. [20] and Tang et al. [10] that CsMPs were one of the possible ^137^Cs carries, derived from decontamination or gust wind. However, the transport mechanism of CsMPs in the resuspension process in the environment was elusive. On the other hand, resuspension of ^137^Cs may be derived from the decontamination process in heavily contaminated areas [21]. Based on abovementioned studies, the resuspension of ^137^Cs and the carriers in the small fluctuation of atmospheric ^137^Cs has not been fully or accurately understood. In particular, it was still found that there was a small fluctuation within 0.0002 Bq∙m^−3^ from January to April and a slightly higher level of atmospheric ^137^Cs from May to September in the aerosol samples obtained in Namie in a heavily contaminated area of Fukushima Prefecture in 2019. Thus, in this study, it is quantitively demonstrated that the carriers of ^137^Cs in early May were the combination of C particles and Al particles (carbon- and aluminum-containing particles; in particular, Al particles dominated at 68%); meanwhile, the predominate carriers of ^137^Cs in late May and September were carbonaceous particles, with an average percentage of 88%. In addition, the effect of weather conditions (precipitation, air temperature, relative humidity, wind speed, and gust-wind speed) on the concentration of ^137^Cs and their carriers was also discussed. Obviously, the temperature and the precipitation were the main impact factors on the distribution of ^137^Cs and its carriers.

## 2. Materials and Methods

### 2.1. Sampling Site

**Figure 1:**
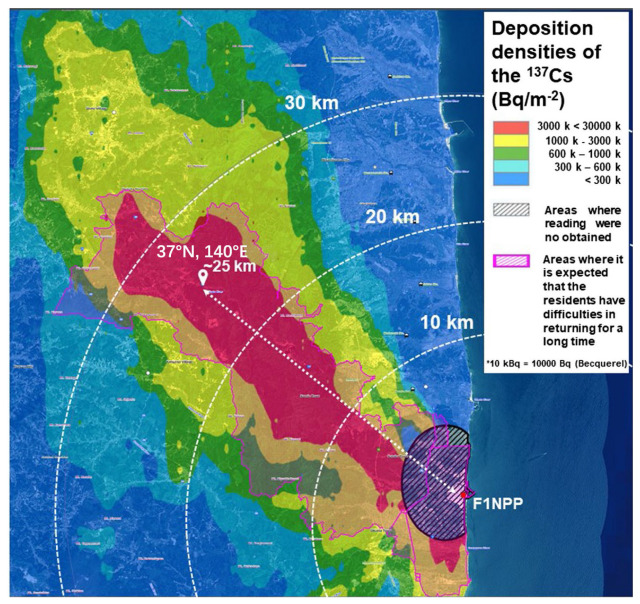
Distribution of the deposited ^137^Cs obtained from the Ministry of Education, Culture, Sports, Science, and Technology (MEXT), converted on 11 March 2013 [22], added with the sampling site in a red cross about 30 km far from FDNPP.

The sampling site was a school ground (37° N, 140° E, about 25 km far from FDNPP) located in a heavily contaminated area of Fukushima Prefecture, as shown in Figure 1. It was surrounded by forests, mainly dominated by deciduous forests. Due to the heavy contamination, residents have been evacuated from these contaminated areas. Therefore, the sampling site was free of residential activities, except for decontamination activities and regular research monitoring. As given in Figure 1, this research site was located at the boundary of the heaviest contaminated area, about 1 MB∙qm^−2^.

### 2.2. Sampling

High-volume aerosol samplers (HV-1000R, Sibata, Saitama, Japan) equipped with quartz fiber filters (2500QAT-UP, Pallflex, Pall, San Diego, CA, USA) were used to collect atmospheric aerosol samples. The sampling flow rate was set to 1000 L∙min^−1^, and the sampling period was a short term of 12 h, as given in the Supplementary Materials. The daytime and nighttime samples were collected in May and September 2019, respectively, with daytime sampling from 6:00 a.m. to 6:00 p.m. and nighttime sampling from 6:00 p.m. to 6:00 a.m. of the next day, as detailed in Table S2. The absent samples in late May and early September 2019 were ascribed to the sampling plan and summer vacation. The quartz filter samples collected by HV aerosol samplers in Namie were stored in the laboratory. In addition, all the sampling information of the aerosol filters is given in Table S3.

### 2.3. Meteorological Monitoring

Regular meteorological monitoring was also carried out at approximately 800 m away from the HV aerosol samplers. The pressure (mbar), solar radiation (W∙m^−2^), moisture content (m^3^∙m^−3^), precipitation (mm), air temperature (°C), relative moisture (RH, %), wind speed (m∙s^−1^), and speed of gust wind (m∙s^−1^) were simultaneously measured per minute. The detailed instrumental settings are shown in Table S4.

### 2.4. Radioactivity Measurement

The radioactivity of ^137^Cs was measured at the peak gamma-ray at 662 keV, using γ-ray spectrometry with an intrinsic germanium semiconductor detector (coaxial type from Ortec EG&G, Eurisys or Canbera, all from Tokyo, Japan) coupled with a computed multichannel analyzer (Oxford-Tennelec Multiport or Seiko EG&G MCA7600, both from Tokyo, Japan). The detection limits of the measurement of ^137^Cs at the Meteorological Research Institute were approximately 9 and 10 mBq per sample, respectively, with a measurement period of 100,000 s, and at Osaka University, they were approximately 14 and 16 mBq per sample, respectively, with a measurement period of 250,000 s. The atmospheric radioactive concentration of ^137^Cs was calculated by

(1) $$C_{filter}=Q_{filter}/V_{filter}$$

where *C_filter_* (Bq∙m^−3^) is the atmospheric radioactive concentration of ^137^Cs, *Q_filter_* (Bq) is the radioactive intensity of ^137^Cs in quartz fiber measured via a coaxial Ge semiconductor, and *V_filter_* (m^3^) is the volume of the sampling air.

### 2.5. Microscope Observations

Two pieces of Φ33 mm were taken out from the HV aerosol filter sample (8 × 10 inches) for the DAPI (4′,6-diamidino-2-phenylindole) staining: one piece (a) was obtained from the middle area, and the other piece (b) was obtained from the edge area from the same aerosol filter sample. The rest of the aerosol filter sample was sealed and stored for other experiments. Then, one piece of Φ12 mm was taken out from each piece of Φ33 mm for further treatment. Finally, two pieces of Φ12 mm were obtained, and the rest of each piece of Φ33 mm was sealed and stored for backup. For all samples, the two pieces of Φ12 mm filters were first fixed by formaldehyde solution and then dried for 2 h. Formaldehyde solution was used for preserving or fixing tissues or cells due to its functions of embalming, fixing cadavers, disinfection, and bleaching. DAPI was used for fluorescent staining because DAPI could penetrate the cell membrane and strongly fix the DNA in the nucleus [23]. After staining, the samples were rinsed with ultrapure water and dried, and in the end, the samples were stored in dark light. For fluorescence observation, the DAPI-stained cells were able to be labeled with blue fluorescence, after excitation by UV light with the wavelength of 360–400 nm, using the fluorescent upright microscope (BS-2040TF, Bio Tools Inc., Gunma, Japan). The dark particles (all particles) could be observed in the reflected-light mode, and the colored particles (carbon-containing particles) could be observed in the fluorescence-mode because the DAPI-stained particles could be shown blue or yellow fluorescence illuminated by UV light under the fluorescent upright microscope. Five sites of each Φ12 mm sample were used for observations using a fluorescent upright microscope. Each site was photographed with a CCD camera in reflected-light mode and in fluorescent-light mode, respectively. Finally, all images were saved for the analysis of the size and morphology of aerosol particles. A total of 10 images were taken for each Φ12 mm sample, and a total of 20 images were taken for each sample collected in our sampling site. The images (the number and Feret’s diameter of particles) were analyzed by a free professional software of ImageJ (version 1.54p) [24]. The detailed treatment process is shown in Figures S1–S5. The morphology, the elemental compositions, and distribution of the aerosol particles were characterized by an imaging plate (IP) system (CR × 25P portable computed radiography, GE Measurement & Control, Billerica, MA, USA) and a SEM (SU3500, Hitachi High-Technologies Co., Tokyo, Japan) equipped with an EDS (X-max, Horiba Ltd., Kyoto, Japan) in MRI under a low vacuum pressure (40 Pa) and with a maximum acceleration voltage of 25 kV. These conditions allowed the quartz fiber filters to be observed by SEM without any pretreatment, such as carbon coating.

## 3. Results and Discussions

### 3.1. Variations in ^137^Cs in Aerosol Filters Sampled in 2019

#### 3.1.1. Annual Variations in Atmospheric ^137^Cs

**Figure 2:**
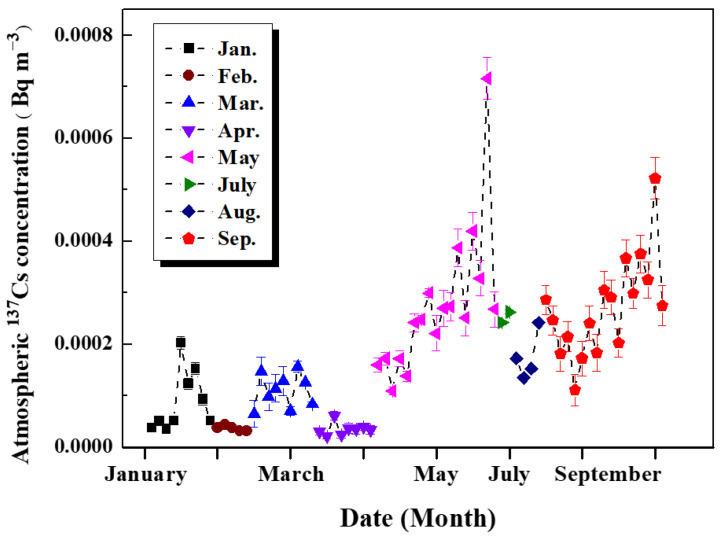
Annual variations in ^137^Cs in aerosol filters collected in 2019.

The annual variations in ^137^Cs in the HV aerosol filters sampled in 2019 are shown in Figure 2 (the absent samples were ascribed to the sampling plan). It could be noticed that there was a small fluctuation of atmospheric ^137^Cs within 0.0002 Bq∙m^−3^ from January to April [16]. It was clear that a slightly higher level of ^137^Cs could be observed from May to September [17]. Moreover, the seasonal variation in atmospheric ^137^Cs showed that the level of ^137^Cs was higher in the warm season (May to September) and lower in the cold season (January to April) [24]. Significantly, there were two peaks that appeared in May (~0.00072 Bq∙m^−3^) and September (~0.00052 Bq∙m^−3^). These two peaks may be due to the resuspension of aerosol particles carrying ^137^Cs, which will be further discussed in detail later. Therefore, in the following section, variations in atmospheric ^137^Cs for HV filter samples collected in May and September are mainly discussed.

#### 3.1.2. Diurnal Variation in ^137^Cs

**Figure 3:**
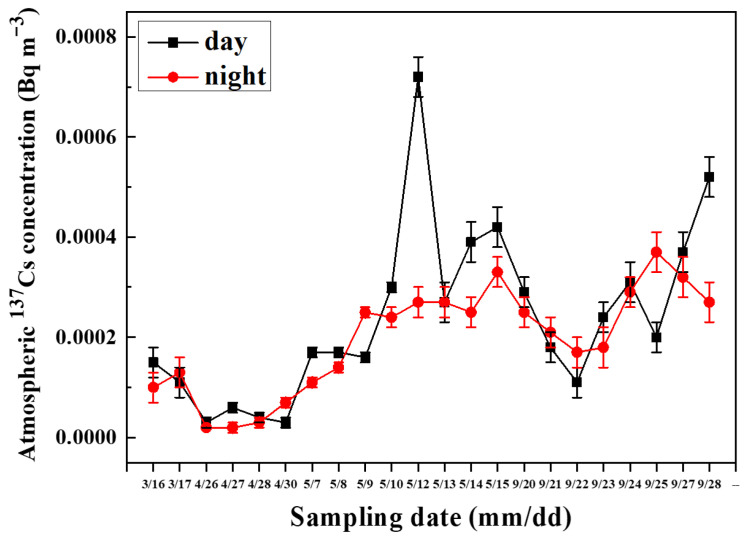
Diurnal variations in ^137^Cs in the aerosol filters sampled in the day period/night period in 2019. The sampling date is defined as mm/dd. Black points represent concentration variations in ^137^Cs in the day period. Red points represent the variations in the samples obtained in the night period.

The comparison of daytime and nighttime variation in atmospheric ^137^Cs is shown in Figure 3. There were 22 groups of ^137^Cs in HV filter samples collected in the day period and night period, respectively. Clearly, it can be found that the concentration of ^137^Cs in the daytime samples was slightly higher than that sampled in the nighttime. Specifically, among the 22 groups of ^137^Cs, 15 groups had a positive difference in atmospheric ^137^Cs between daytime and nighttime samples. Also, it can be noticed that the concentrations of ^137^Cs sampled in the day period on 12 May and on 28 September were about three times and two times higher than those collected during the night period, respectively. In particular, the diurnal variations in ^137^Cs were similar to the seasonal variation in ^137^Cs, as mentioned above in the annual variations in ^137^Cs. Specifically, the maximum of ^137^Cs concentration was 0.00072 Bq∙m^−3^ sampled in a day period on 12 May, and the minimum was 0.00002 Bq∙m^−3^, sampled in the night period on 26 April and 27 April. In autumn (late September), the maximum of ^137^Cs concentration was 0.00052 Bq∙m^−3^ sampled during a day period on 28 September, and the minimum was 0.00011 Bq∙m^−3^ sampled during a day period on 22 September.

### 3.2. Carriers of ^137^Cs in May and September

#### 3.2.1. Carriers of ^137^Cs for the HV Samples Collected in May 2019

**Figure 4:**
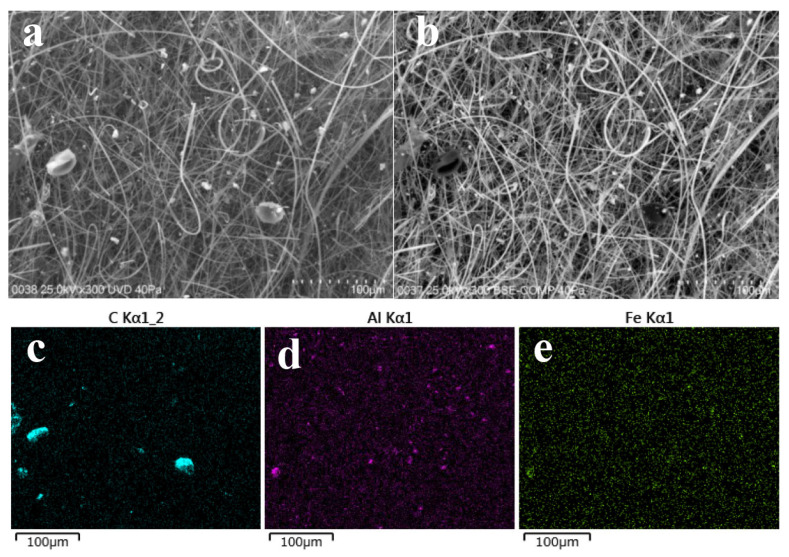
SEM images and elemental distribution maps of the HV filter named #NHVA2019-0511-J-Q: (**a**) SEM image obtained in backscattered electrons mode; (**b**) SEM image obtained in low-vacuum mode; (**c**) elemental distribution of carbon; (**d**) elemental distribution of aluminum; and (**e**) elemental distribution map of iron.

SEM observations of the HV filter sample (named as #NHVA2019-0511-J-Q) are shown in Figure 4. Comparative analysis of the observations is shown in Figure 4a in back-scattered electron mode (BSE), and in Figure 4b in low-vacuum mode. Clearly, there were some grayish-white particles with diameters of 20–35 μm that could be easily identified as pollen particles. Meanwhile, there were also some small, clear, white, elliptic-shaped particles, and they were mapped by EDS, as shown in Figure 4b–d. Significantly, the several large carbon-containing particles could be pollens, as shown in Figure 4c, and some small particles may be organic matters (such as fungal cells and/or debris, sporangia, ascospores, or other microorganisms). In Figure 4d,e, it can be found that there were a lot of small aluminum-containing and iron-containing particles, which may be mineral particles or soil dust. Overall, in spring, more iron/aluminum-containing mineral particles of 2–5 μm and some scattered pollens and/or organic particles can be observed, indicating that the main possible carriers of ^137^Cs in the HV filters collected were the mineral particles.

**Figure 5:**
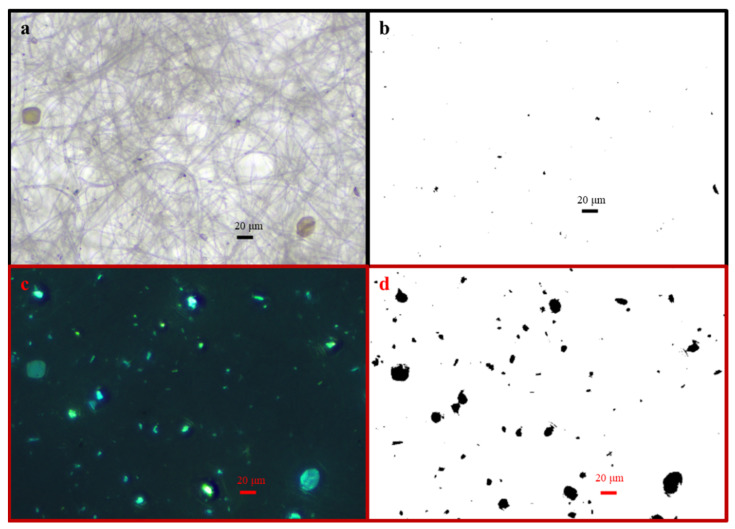
Microscope images (BS-2040TF) and processed equivalent projected images by ImageJ in the same site of an HV filter sample (#NHVA2019-0523-J-Q) collected in May 2019: (**a**) microscope image in reflected-light mode; (**b**) equivalent projected area image of (**a**); (**c**) microscope image in fluorescent-light mode; and (**d**) equivalent projected area image of (**c**).

In the typical optical microscopy photograph (Figure 5a) and fluorescent micrograph (Figure 5c) of 4,6-diamidino-2-phenylindole (DAPI) staining particles in the HV filter sample (#NHVA2019-0523-J-Q), there are a lot of bioaerosol particles found. After treatment by ImageJ software, equivalent projected images were used for counting and classifying particles in the reflected-light mode (Figure 5b) and fluorescent-light mode (Figure 5d), respectively. It is easy to distinguish some pollen particles with a size larger than 20 μm (Figure 5a). Based on the fluorescent color and morphology of the lighted particles, the fluorescent aerosol particles could be classified into different bioparticles. Specifically, in Figure 5c, it can be found that the most abundant fluorescent aerosol particles are (1) big elliptic blue particles (diameter > 20 μm, indicating pollens or aggregated particles); (2) spindly yellow and blue particles (10 μm < diameter < 20 μm, microbial particles of sporangia or ascospores); (3) elliptic yellow and blue particles (diameter < 10 μm, identified as bacteria or basidiospore); and (4) white particles, indicating other organics.

**Figure 6:**
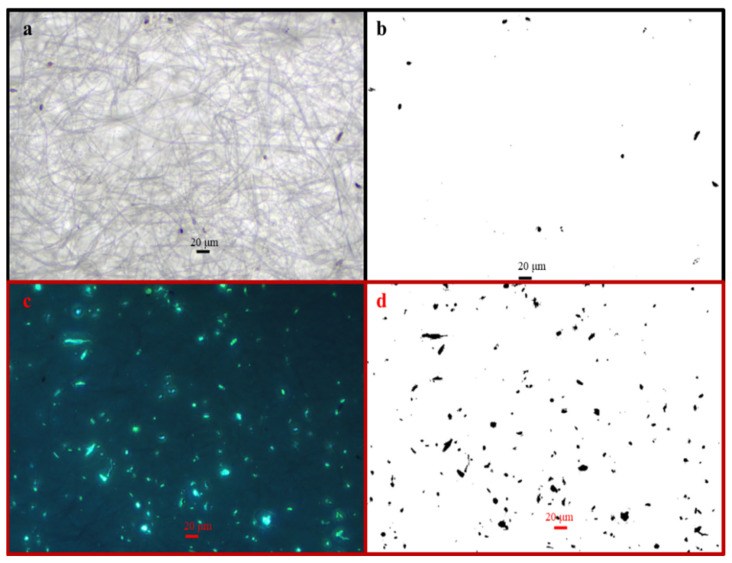
Microscope images (BS-2040TF) and processed equivalent projected images by ImageJ in the same site of an HV filter sample (#NHVA2019-0923-L-Q) collected in September 2019: (**a**) microscope image in reflected-light mode; (**b**) equivalent projected area image of (**a**); (**c**) microscope image in fluorescent-light mode; (**d**) equivalent projected area image of (**c**).

The classifications of fluorescence-highlighted particles were consistent with those in Reference [17]. Additionally, only particles with sizes larger than 0.65 μm were counted in this study. Particularly, we observed numerous particles with multiple septa that were most possibly the fungal spores of the phylum Ascomycota. More bioaerosol particles were observed in September than in May (based on the comparation in Figure 5c and Figure 6c). It was possibly due to a seasonal change in the bioaerosol source or rainy weather on the sampling days in September, as has also been discussed in the report of Kita et al. [23]. As a consequence, after analyzing all particles, large particles (such as ascospores, pollens, fragments, and aggregated particles), small particles (such as bacteria), and medium particles (such as basidiospores) could be observed and classified. Therefore, the abovementioned six types of particles are discussed in the following section.

Based on the SEM observations (Figure 4), it could be obtained that many small mineral particles were also found in HV filter samples collected in May. Meanwhile, the optical microscope images (Figure 5) also exhibited many dispersed organic large particles, such as pollen particles. Thus, it was assumed that the carriers of ^137^Cs in the filter samples collected in May can be alternated during this sampling period, because May was just located in the alternate period of spring and summer [15]. In addition, four HV filter samples collected in May were analyzed by SEM/EDS in order to estimate the elemental mass percentage. The variations in ^137^Cs and elemental mass percentage of Al and C with sampling time are shown in Figure S6. The black points are the concentration of ^137^Cs, and the red and the blue symbols represent the percentage of C and Al, respectively. It was obvious that the variation in ^137^Cs concentration was consistent with the trend of Al% [16]. Specifically, the gradual increase in ^137^Cs concentration and Al% was observed from 26 April to 12 May, reaching the peak on 12 May. After 12 May, Al% showed a clear downward trend. In contrast, the variation in C% always had a slowly increasing trend. There would be a period for ^137^Cs carriers’ transition from mineral particles (aluminum-containing particles) to carbonaceous particles. Therefore, it could be assumed that mineral particles or soil dust could be the main carriers of ^137^Cs in early spring [11]. On the other hand, the carbon-containing particles may be the dominated carriers of ^137^Cs in late spring, but more data and further discussion are still needed [14].

In Figure S7, the comparative variation in carbon-containing particles and aluminum-containing particles estimated from SEM-EDS observations is provided. It can be noticed that the percentage of carbon-containing particles gradually increased; on the contrary, the percentage of aluminum-containing particles gradually decreased from 10 May to 15 May, which is consistent with the assumption that the mineral particles or soil dusts could be the main carriers of ^137^Cs in early spring, and the carbon-containing particles could be the dominated carriers of ^137^Cs in late spring. Moreover, a close higher-level percentage of carbon-containing particles was found in the samples collected on 15 May and 23 September, with the percentage of carbon-containing particles being 92% and 82%, respectively, indicating that the main carriers of ^137^Cs may be carbon-containing particles. This result was consistent with our previous master’s research [24], which postulated that the bioparticles gradually became the dominant carriers of ^137^Cs. These results were also consistent with the speculation that the mineral particles or soil dusts could be the main carriers of ^137^Cs in early spring, and the carbon-containing particles could be the gradually dominated carriers of ^137^Cs in late spring.

#### 3.2.2. Carriers of ^137^Cs for the HV Samples Collected in September 2019

As shown in the typical optical microscopy photograph (Figure 6a) and fluorescent micrograph (Figure 6c) of DAPI staining particles in the HV filter sample (#NHVA2019-0923-L-Q), it was easily observed that there were more bioparticles with different morphology and size, a finding consistent with reports that bioparticles could be the main carriers of ^137^Cs in autumn [17,25].

**Figure 7:**
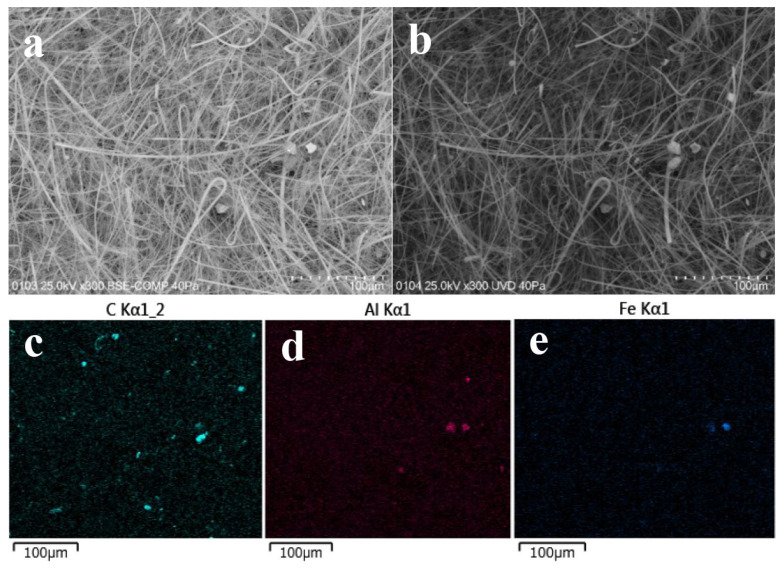
SEM images and elemental distribution maps: (**a**) SEM image obtained via BSE model; (**b**) SEM image obtained via UVD; (**c**) compositional map of C; (**d**) compositional map of Al; and (**e**) compositional map of Fe. Sample name is #NHVA2019-0929-B-Q. The bar is 100 μm.

SEM observations of the HV filter sample (#NHVA2019-0929-B-Q) were shown in Figure 7. Compared with Figure 7c–e, there were a higher level of carbonaceous particles and fewer aluminum-containing and iron-containing particles, which indicates carbon-containing particles could be the dominated carriers of ^137^Cs in September. It was also consistent with the results of microscope observations, as shown in Figure 6.

### 3.3. Bioaerosol Particles and Their Size Distributions

According to the observations of optical, fluorescent microscope, and SEM-EDS, aerosol particles in the aerosol filter samples collected in May and September 2019 were mainly analyzed. Diameter was described as the Feret diameter (along with the selection boundary, the longest distance between any two points, also known as maximum caliper), which was obtained from the microscope images using ImageJ. Figure S8a,b were the size distributions of bioaerosol particles in the HV filter samples collected in May 2019. It could be easily noticed that the particles with the diameter (*d* < 1 μm) were predominant. The second peak represented the particles with a diameter less than 2 um. Similarly, the size distribution of bioaerosol particles in the HV filter samples collected in September 2019 is given in Figure S8c. A wider diameter range of the bioaerosol particles could be found in three peaks (*d* < 1 μm, *d* < 2 μm, and *d* < 8 μm). In addition, bioaerosol particles with a diameter less than 1 μm were also predominant. Quantitatively, from Figure S8a (early May) to Figure S8b (late May), the normalized number of particles increases nearly three times (obtained by the value of first peak), which is consistent with the SEM observations, as shown in Figure S8. Similarly, from Figure S8b (late May) to Figure S8c (September), the normalized number of particles has no apparent change. Overall, although the size distribution appears bimodal or multi-peaked in Figure S8b,c, the bioaerosol particles with the diameter (<2 μm) could be the predominant possible carriers of ^137^Cs.

### 3.4. Relation Between Aerosol Particles and Atmospheric ^137^Cs

In Figure S9a, the unstained particles represent mainly mineral particles and some bioaerosol particles, which were difficult to stain in the DAPI staining experiment. In Figure S9b, the stained particles refer to mainly bioaerosol particles, which could be observed in blue/yellow/white-lighted particles under fluorescent light. The blue points and the red points represent the correlation between the concentration of atmospheric ^137^Cs and the concentration of aerosol particles, which were estimated from the HV aerosol filter samples collected in May 2019 (there were ten samples, *n* = 10) and in September 2019 (there were eight samples, *n* = 8), respectively. It was obviously found that the unstained particles of mainly mineral particles had a strong positive correlation with the concentration of atmospheric ^137^Cs both in May and September 2019, as shown in Figure S9a. As mentioned in Figure 6, Figure 7, Figures S7 and S8, it was consistent with the assumption that the combinations of mineral particles and bioparticles could be the main possible carriers of ^137^Cs in May 2019.

In Figure S9b, the stained particles also had a positive correlation distribution with ^137^Cs in September 2019, reconfirming that the bioparticles could be predominant carriers of ^137^Cs (which was also consistent with the results in Figure 6 and Figure 7). In contrast, in May, the ^137^Cs concentration did not have a good linear relation with concentration of colorless particles, and this may be caused by the fact that the combinations of mineral particles and bioparticles could be the main carriers of ^137^Cs in May 2019. Namely, the dominant carriers of ^137^Cs could be mineral particles in early May and bioparticles in late May (as shown in Figure 5 and Figure S7). It seems to be consistent with other studies [16,17] that there were different resuspension mechanisms in May and September. Namely, it was generally believed that there was a much lower concentration of ^137^Cs, and the main carriers could be mineral particles in the spring. In summer and autumn, there were relatively higher concentrations of ^137^Cs, and the bioaerosol particles could be predominant in the aerosol particles, which also implied that the bioaerosols were more possible to be the carriers of ^137^Cs in September [24].

Igarashi et al. reported a strong relationship between carbon-bearing particles and ^137^Cs concentration [17]. Also, combining the abovementioned discussion, the main carriers of ^137^Cs were mineral particles in early May, and the predominant carriers of ^137^Cs were bioaerosol particles in late May and in September 2019. Therefore, it was necessary to quantify the relation between bioaerosol concentration and ^137^Cs concentration to estimate the predominant contribution of the specific type of bioaerosol particles to the atmospheric ^137^Cs. According to the classification, as shown in Table S5, there were several common types of bioaerosol particles [26,27,28,29]. Then, a multiple linear regression was used to estimate the kinds of bioparticles variated with the dominant carriers of ^137^Cs, as follows:

(2) $$I_{j}=\sum_{i,j}a_{i}\times A_{i,j}+b_{0}$$

where *I_j_* (Bq) is the total radioactivity of ^137^Cs in the HV aerosol filter samples; *j* represents the observed 10 samples containing predominant bioaerosol carriers of ^137^Cs in late May and in September 2019; *i* = 1, 2, 3, 4, 5, 6 (little particle, bacteria, spore, ascospore, pollen, and fragment), and so on; *a_i_* is the coefficient (Bq∙μm^−2^); *A*_i_ is the area of the HV filter samples (183.2 mm × 234 mm); and *b*_0_ is the residual radioactivity of ^137^Cs of the HV filter (Bq). Each coefficient in Equation (2) is estimated in Table S6.

In Figure S10, the relationship between each type of bioaerosol and ^137^Cs was obtained via multiple linear regression analysis. The black symbol is the measured value of ^137^Cs obtained from the coaxial Ge semiconductor detector. The red points represent the predicted radioactivity value estimated from Equation (2). The other symbols represent the contribution of different bioaerosol particles to the radioactivity of ^137^Cs.

The distribution of ^137^Cs for each bioaerosol in aerosol filter samples collected in 2019 is given in Table S7. As a result of a multivariate analysis (performed by least-squares) under non-negative constraints, the contribution of each species of bioaerosols to the radioactivity of ^137^Cs and the residuals was calculated. Obviously, bacteria (blue squares) had the highest contribution to the radioactivity of ^137^Cs (Figure S10), which showed the strongest correlation with the concentration of ^137^Cs, followed by the order of little particles (wine squares), spores (violet squares), and ascospores (magenta squares), and fragment (olive squares). It was strongly consistent with the results of the size distribution of particles (as shown in Figure S8c), in which particles with a diameter (<2 μm) could be the predominant possible carriers of ^137^Cs. Therefore, combining the information on the size distribution of the particles (Figure S8), the dominant ^137^Cs-bearing particles could be bacteria (1–1.8 µm), followed by little particles (less than 1 µm) and medium particles of 1.8–10 µm, possibly derived from spores. In addition, to verify the accuracy of Equation (2), a correlation analysis between the observed and estimated radioactivity values was conducted, and the results are shown in Figure S11. The *x*-axis is the observed value of the atmospheric radioactivity of ^137^Cs, and the *y*-axis is the estimated value of the atmospheric radioactivity of ^137^Cs. It is obvious that they have a good, positive linear relationship, indicating the feasibility and reasonability of Equation (2).

### 3.5. Effect Estimation of Weather Conditions on Atmospheric ^137^Cs and Its Carriers

Several representative items of weather conditions, such as precipitation, air temperature, relative humidity (RH), wind speed, and gust-wind speed, were selected and monitored to analyze the influence of these weather factors on the concentration of atmospheric ^137^Cs and its carriers, as shown in Table S8. Precipitation and other values of air temperature, relative humidity, and wind speed were the average value, as well as the gust speed (was the maximum sudden increase in wind speed above the average wind speed) obtained from a 24 h accumulated value during the sampling period. It could be found that the precipitation had a negative effect on both the concentration of ^137^Cs and its carriers. The temperature had a positive effect on the concentration of ^137^Cs and its carriers, a result which was consistent with the abovementioned speculation that the concentration of ^137^Cs was higher in the warm season and lower in the cold season. Other weather conditions, namely RH, wind speed, and gust, had no significant correlations in the current research.

As we know, it is easy to understand that the precipitation was conducive to washing away ^137^Cs particles in the atmosphere, resulting in a decrease in both atmospheric ^137^Cs concentration and ^137^Cs particles, referred to as wet deposition. Additionally, it was demonstrated that wet deposition was also one of the main mechanisms for removal of particulate matters and organic pollutants from the atmosphere, resulting in low fluctuation of atmospheric ^137^Cs [29]. In contrast, precipitation had a negative impact on the concentration of ^137^Cs and a positive effect on the concentration of the particles in September, suggesting that the particle-generation processes stimulated by precipitation were more significant than those of wet deposition. This observation was consistent with the fact that the precipitation was conducive to an increase in the atmospheric ^137^Cs compared with the non-rainfall sampling period by generating more bioaerosol particles [23]. In September, because the rainfall is beneficial for the reproduction of spores, resulting in a positive correlation between the precipitation and the concentration of the bioparticles Moreover, there was no doubt that higher temperature could accelerate microbial colonization, which was able to explain the positive impact on the concentration of ^137^Cs and its carriers by temperature both in May and September.

## 4. Conclusions

According to the annual atmospheric ^137^Cs variations obtained from the HV aerosol filter samples collected in 2019, a small fluctuation within 0.0002 Bq∙m^−3^ from January to April and a slightly higher level of ^137^Cs can be observed from May to September. Therefore, it could be obtained that the concentration of ^137^Cs was higher in the warm season (May to September) and lower in the cold season (January to April). Significantly, based on observations of SEM/EDS and optical microscope, it could be quantitatively determined that the small mineral particles or soil dusts were the main carriers of ^137^Cs in early spring, and the carbon-containing particles (some microorganisms, spores, and some other bioparticles) were the dominate carriers of ^137^Cs late spring; meanwhile, the bioparticles (bacteria and other small bio-aerosols particles) were dominant carriers of ^137^Cs in September. Moreover, it could be obtained that the main impact factors of ^137^Cs and its carriers were the temperature and the precipitation among precipitation, air temperature, RH, wind speed, and gust. Specifically, the temperature had a positive effect on the concentration of ^137^Cs and its carriers both in May and September 2019, an observation that is consistent with the abovementioned speculation that ^137^Cs concentration was higher in the warm season and lower level in the cold season [16]. Additionally, the rainfall had a negative effect on both the concentration of ^137^Cs and its carriers in May 2019. In contrast, the rainfall had a negative impact on the concentration of ^137^Cs and a positive effect on the concentration of the particles in 2019, which may result from the fact that the effect of precipitation on the concentration of ^137^Cs and/or bioparticles may persist for some time. For instance, the rainfall in one day may affect changes in the concentration of ^137^Cs and/or bioparticles in the following several days.

Significantly, there was no decreasing trend in the concentration of atmospheric ^137^Cs [30], meaning that long-term monitoring was still necessary for further and deeply understanding the fate and variation in atmospheric ^137^Cs resuspended from ^137^Cs-bearing microparticles [10,31,32,33] or soluble ^137^Cs [34] derived from FDNPP. Also, it was difficult to identify or observe individual ^137^Cs-containing bioparticles in this study, which opened a new insight into the development of relative techniques to directly observe individual ^137^Cs-containing bioparticles for further future research.

## Data Availability

The original contributions presented in this study are included in the article/Supplementary Materials. Further inquiries can be directed to the corresponding author.

## Supplementary Materials

The following supporting information can be downloaded at https://www.mdpi.com/article/10.3390/toxics14010088/s1, Figure S1: The observations of aerosol filters (NHVA-20190124-Imp-Q and HVA-20190523-Ura-Q) by imaging plate system (CR × 25P portable computed radiography, GE Measurement & Control, Massachusetts, USA); Figure S2: One new aerosol filter (8 × 10 inches) was used in HV aerosol sampler in 2019; Figure S3: Ten observation sites in each aerosol filter sample. The treatment process was given as follows: (1) there was an HV air filter sample with an area of 183.2 mm × 234 mm, as shown in the grayish-white rectangle; (2) 2 pieces of Φ33 mm samples were taken out of this aerosol filter (a was located at the center of aerosol filter; b was located at an edge of quartz filter, as shown in light-blue circles); and (3) five observation sites were located on each orange circle (as shown in red points). Thus, each sample needs to be photographed separately in reflected-light mode and fluorescent-light mode with a CCD camera. Totally, 20 images were observed and analyzed for each aerosol filter sample; Figure S4: Experimental process of DAPI staining procedures: (a) the process of adding 250 μL of 1% formalin solution to the filter holder equipped with one piece of Φ12 mm filter sample and leaving it for 1 h; (b) the process of adding 200 μL of ultrapure water for rinsing the sample; (c) the process of adding 10 μL of DAPI solution and then leaving it for 15 min; and (d) the process of adding 200 μL of ultrapure water for rinsing, and then drying for 2 h. Finally, the stained Φ12 mm filter sample was removed, placed on a glass slide, stored in the dark, and subjected to observation under the optical microscope; Figure S5: Microscope images (BS-2040TF) and processed equivalent projected images by ImageJ in the same site of an HV filter sample (#NHVA2019-0923-L-Q) collected in September 2019: (a) microscope image in reflected-light mode; (b) equivalent projected area image of Figure S5a; (c) microscope image in fluorescent-light mode; and (d) equivalent projected area image of Figure S5c; Figure S6: Variation in ^137^Cs, C%, and Al%; Figure S7: Comparative percentage variations in carbon- and aluminum-containing particles; Figure S8: Normalized particle number size distributions of bioaerosol particles in the HV filter samples collected in May (a: #NHVA2019-0501-G-Q; b: #NHVA2019-0523-J-Q) and September (c, #NHVA2019-0923-J-Q) in 2019; Figure S9: The concentration variations in atmospheric ^137^Cs with the concentration of aerosol particles, which were respectively estimated from the HV aerosol filter samples collected in May 2019 (there were ten samples, n = 10, highlighted in blue points) and in September 2019 (there were eight samples, n = 8, highlighted in red points); Figure S10. Bioaerosol particles’ radioactivity contribution to ^137^Cs radioactivity based on the multiple linear regression Equation (2). The black squares are the measured values (*I*) of ^137^Cs radioactivity in each HV aerosol filter sample. The red squares represent the estimated values (*I*) from Equation (2) in each sample. The rest of the symbols represent the contributed radioactivity of different bioaerosol particles to the radioactivity of ^137^Cs based on statistical predictions from the multiple linear regression equation; detailed information is summarized in Table S5; Figure S11: Correlation between observed values and estimated values for ^137^Cs radioactivity; Table S1: Radionuclides released from FDNPP accident modified from report of Ohara et al. [5]; Table S2: The information of samples in May and September 2019, giving the name and the sampling time; Table S3: All sampling information of the aerosol filters; Table S4: Several instrumental specifications of meteorological monitoring modified from Appendix of Ishizuka et al. [15]; Table S5: Classification of bioaerosol particles based on different sizes. AR (aspect ratio) represents the ratio of the major to the minor. The major and the minor are the primary and secondary axes of the best-fitting ellipse. Circularity is defined as 4π × (area)/(squared circumference). A value of circularity of 1 represents the particle that could be regarded as a perfect circle. If the value approaches 0, it indicates that the shape of the particle is elongated. When the AR is greater than 4 and the circularity is less than 0.45, the particle is defined as a fragment; Table S6: Each coefficient in Equation (2). *a_i_* is the coefficient of each bioaerosol, referring to the ^137^Cs radioactivity accumulated in each type of bioaerosols within each HV aerosol filter sample. The statistical analysis was made by regression analysis for 10 samples. Asterisk (⁎) indicates a significant level; Table S7: Contribution values of each species of bioaerosols to ^137^Cs radioactivity. There were ten data (eight in September, and two on 15 May, consideration of bioaerosols as the main carriers). The observed values (Bq) of ^137^Cs radioactivity are obtained from the HV aerosol filter samples (183.2 mm × 234 mm). The estimated values (Bq) are based on a prediction of Equation (2) in each sample. The predicted radioactivity (Bq) in little particles, bacteria, spores, ascospores, and fragments (without considering pollens because of seasonality) was calculated from statistical analysis in multiple linear regression Equation (2). The residual values were involved in the difference between the measured value and the estimated value; Table S8: The variation in atmospheric ^137^Cs concentration, precipitation, temperature, relative humidity, wind speed, and gust collected at sampling location.
