# Supplementary material for: Variation in Atmospheric 137Cs and the Carriers in Aerosol Samples Obtained from a Heavily Contaminated Area of Fukushima Prefecture
Source: Toxics. 2026 Jan 19;14(1):88. doi: 10.3390/toxics14010088 (PMC12845861; doi:10.3390/toxics14010088)
Supplement: Supplementary file 1 [file toxics-14-00088-s001.zip › toxics-4089600-supplementary.pdf]

Variation in atmospheric ^{137}Cs and the carriers in aerosol samples obtained from a heavily contaminated area of Fukushima prefecture

Huihui Li[1], Peng Tang[1, 2*], Kazuyuki Kita[3,*]

1. School of Chemical Engineering, Sichuan University of Science and Engineering, Zigong, Sichuan, 643000, China.

2. Tianjin Key Laboratory of Brine Chemical Engineering and Resource Eco-utilization, College of Chemical Engineering and Materials Science, Tianjin University of Science and Technology, No.29, 13th Street, Binhai New District, Tianjin Municipality 300457, China.

3. Graduate School of Science and Engineering, Ibaraki University, 2-1-1 Bunkyo, Mito, Ibaraki, 310-8512, Japan.

*Correspondence: tpdzyyx@163.com; kazuyuki.kita.iu@vc.ibaraki.ac.jp

1. Methods

1.1 Counting the numbers of the particles

As reported in the previous work of the Kita group (Igarashi et al., 2019; Kita et al., 2020), the optical microscopy photos of HV filter samples (each red point, as shown in Figure S3) were analyzed by ImageJ software for the counting of the numbers of the particles: (i) adjust the "Saturation" to find faintly colored particles in the photo obtained in the reflected-light mode (see Figure S5b), counting the numbers (n_i) of particles (see Figure S5a); (ii) adjust the "Brightness" and "Contrast" to find thick, dark-colored particles in the

observation observed under the fluorescence-mode (see Figure S5d), counting the numbers (n_{ii}) of colored particles (see Figure S5c); and (iii) record the total numbers ($n = n_i + n_{ii}$) of the particles in each observation site.

1.2 Sampling introduction

High-volume aerosol samplers (HV-1000R, Sibata, Japan) equipped with quartz fiber filters (2500QAT-UP, Pallflex, USA) were used to collect atmospheric aerosol samples. All sampling information of the aerosol filters is shown in Tables S2 and S3. The sampling flow rate was set to 1000 $Lmin^{-1}$, and the sampling period was a short term of 12 hours. The daytime and nighttime samples were respectively collected in May and September 2019, with day-time sampling from 6:00 a.m. to 6:00 p.m., and night-time sampling from 6:00 p.m. to 6:00 a.m. of the next day, as detailed in Table S2. In all sampling processes, aerosol filter samples were collected at a height of 1.2 m above the ground. The collected filter samples were stored in the laboratory.

2. Supplementary Figures and Tables

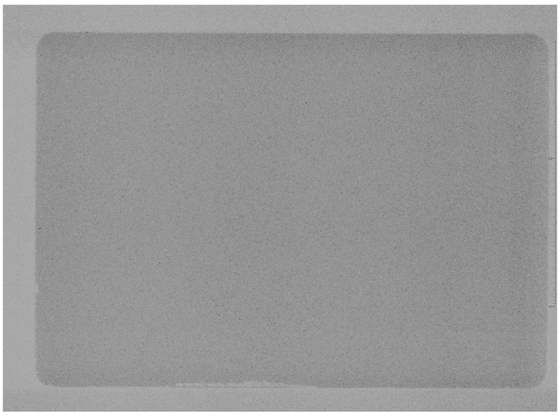

Aerosol filter, NHVA-20190124-Imp-Q with ^{137}Cs concentration of 1.23×10^{-4} Bq/m^3 without identification of particle bearing ^{137}Cs

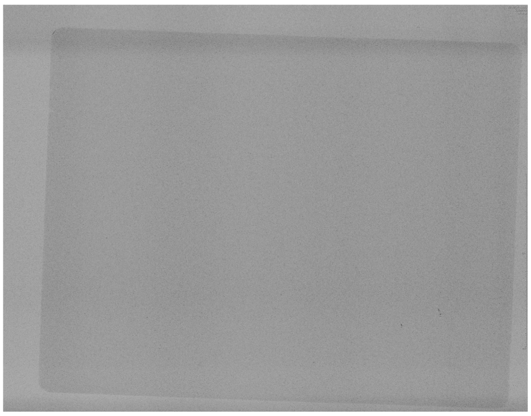

Aerosol filter, NHVA-20190523-Ura-Q with ^{137}Cs concentration of 7.16×10^{-4} Bq/m^3 without identification of particle bearing ^{137}Cs

Figure S1. The observations of aerosol filters (NHVA-20190124-Imp-Q and HVA-20190523-Ura-Q) by imaging plate system (CR×25P portable computed radiography, GE Measurement & Control, Massachusetts, USA).

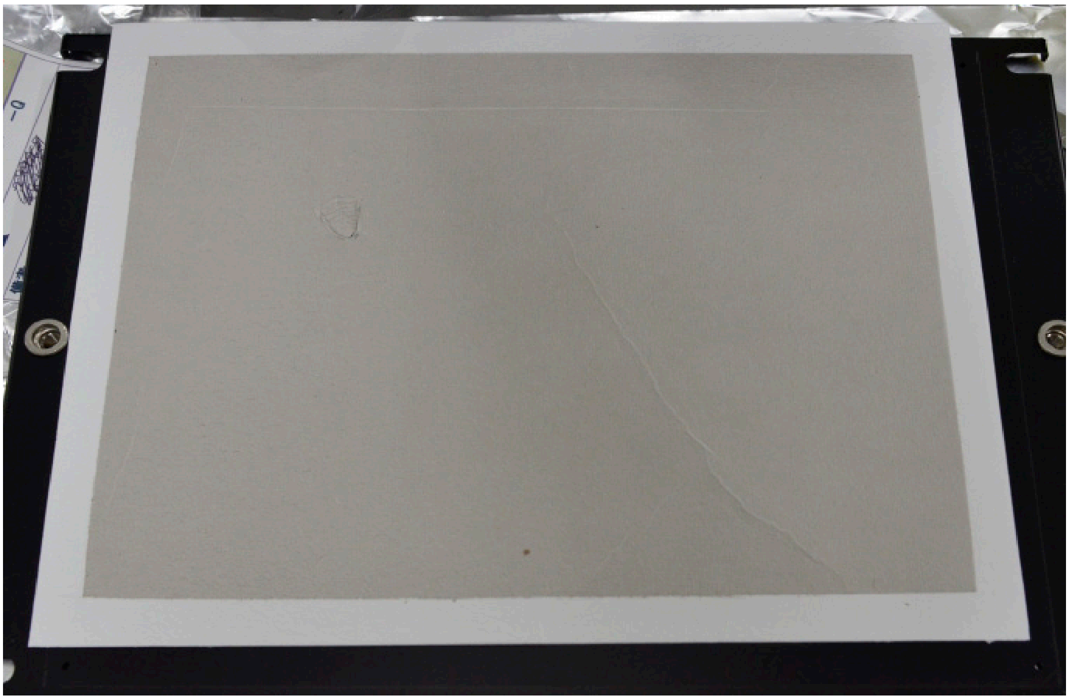

Figure S2. One new aerosol filter (8× 10 inches) was used in HV aerosol sampler in 2019.

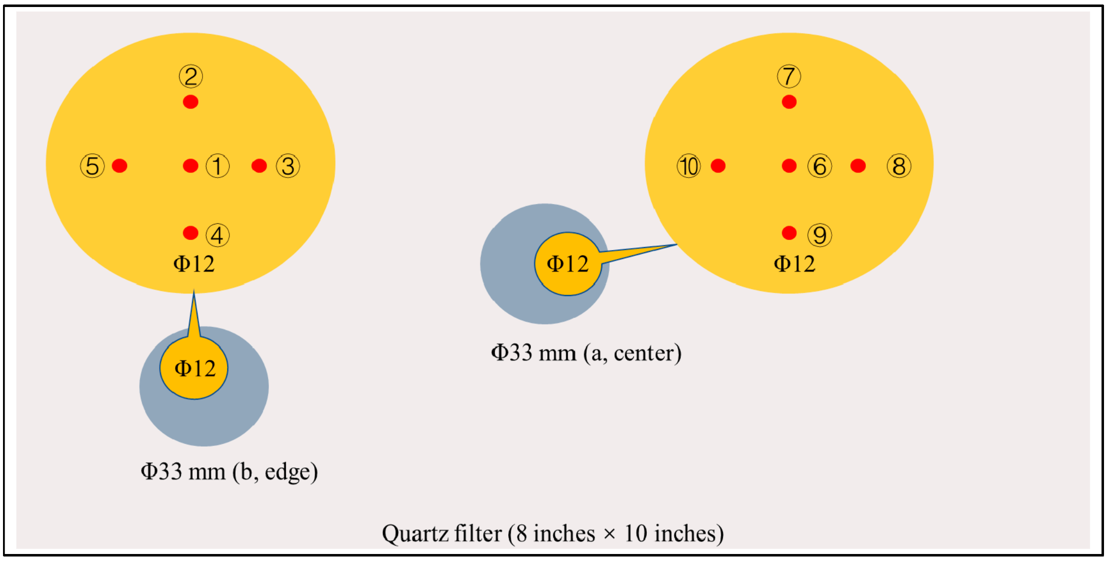

Figure S3. Ten observation sites in each aerosol filter sample. The treatment process was given as follows: (1) there was an HV air filter sample with an area of 183.2 mm × 234 mm, as shown in the grayish-white rectangle; (2) 2 pieces of Φ33 mm samples were taken out of this aerosol filter (a was located at the center of aerosol filter; b was located at an edge of quartz filter, as shown in light-blue circles); (3) five observation sites were located at each orange circle (as shown by red points). Thus, each sample needs to be photographed separately in reflected-light mode and fluorescent-light mode with a CCD camera. Totally, 20 images were observed and analyzed for each aerosol filter sample.

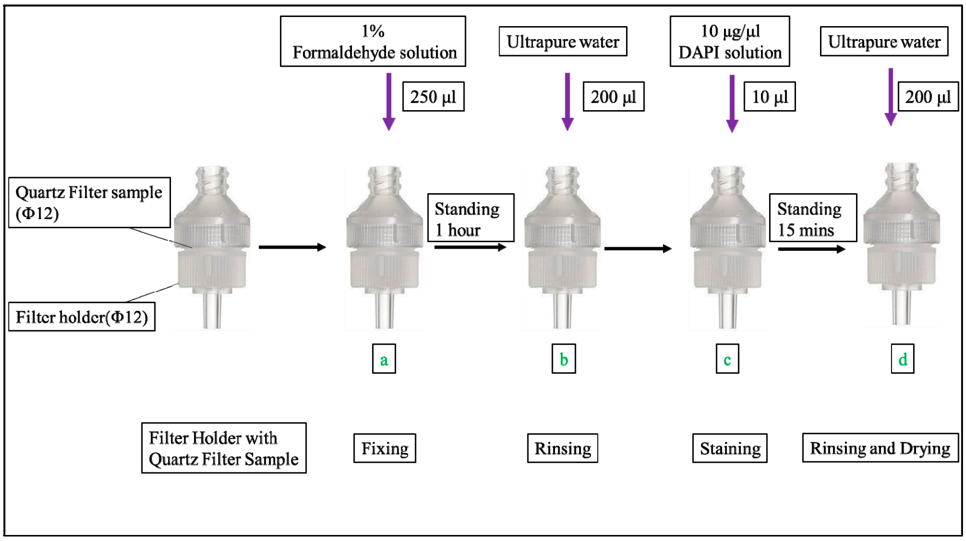

Figure S4. Experimental process of DAPI staining procedures: (a) the process of adding 250 μL of 1% formalin solution to the filter holder equipped with one piece of Φ12 mm filter sample and leaving it for 1 hour; (b) the process of adding 200 μL of ultrapure water for rinsing the sample; (c) the process of adding 10 μL of DAPI solution and then leaving it for 15 minutes; and (d) the process of adding 200 μL of ultrapure water for rinsing, and then drying for 2 hours. Finally, the stained Φ12 mm filter sample was removed, placed on a glass slide, stored in the dark, and subjected to observation under the optical microscope.

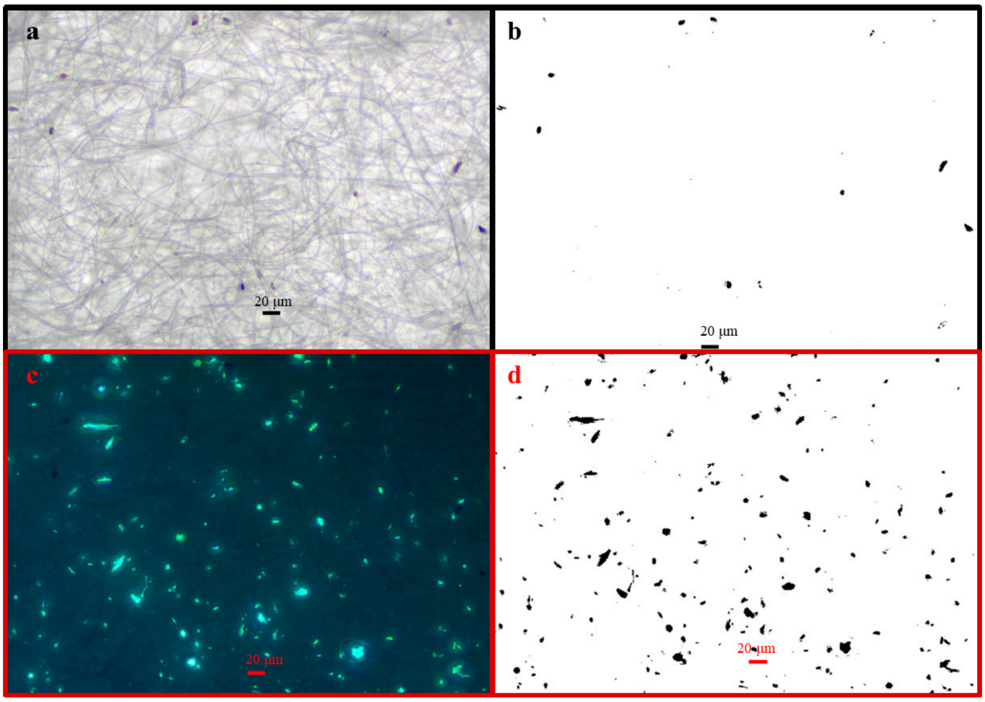

Figure S5. Microscope images (BS-2040TF) and processed equivalent projected images by ImageJ in the same site of an HV filter sample (#NHVA2019-0923-L-Q) collected in September 2019: (a) microscope image in reflected-light mode; (b) equivalent projected area image of Figure S5a; (c) microscope image in fluorescent-light mode; and (d) equivalent projected area image of Figure S5c.

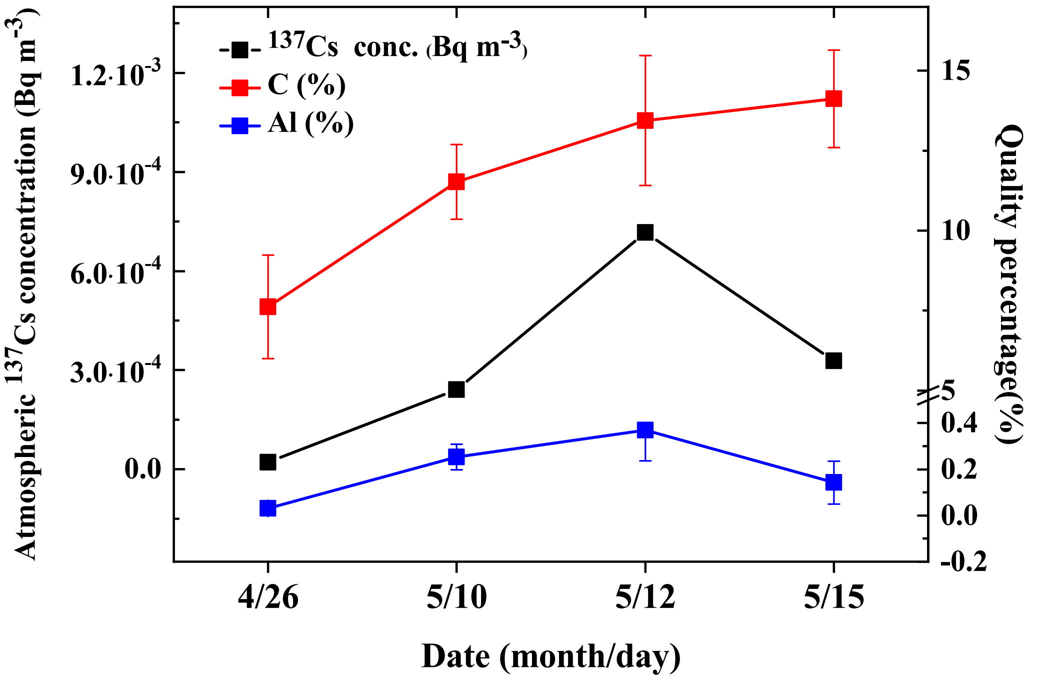

Figure S6. Variation in ^{137}Cs, C%, and Al%.

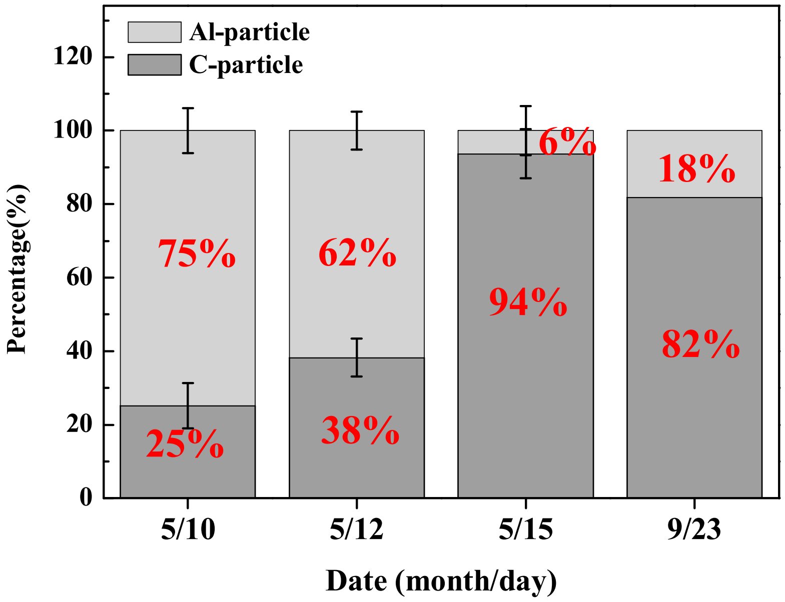

Figure S7. Comparative percentage variations in carbon- and aluminum-containing particles.

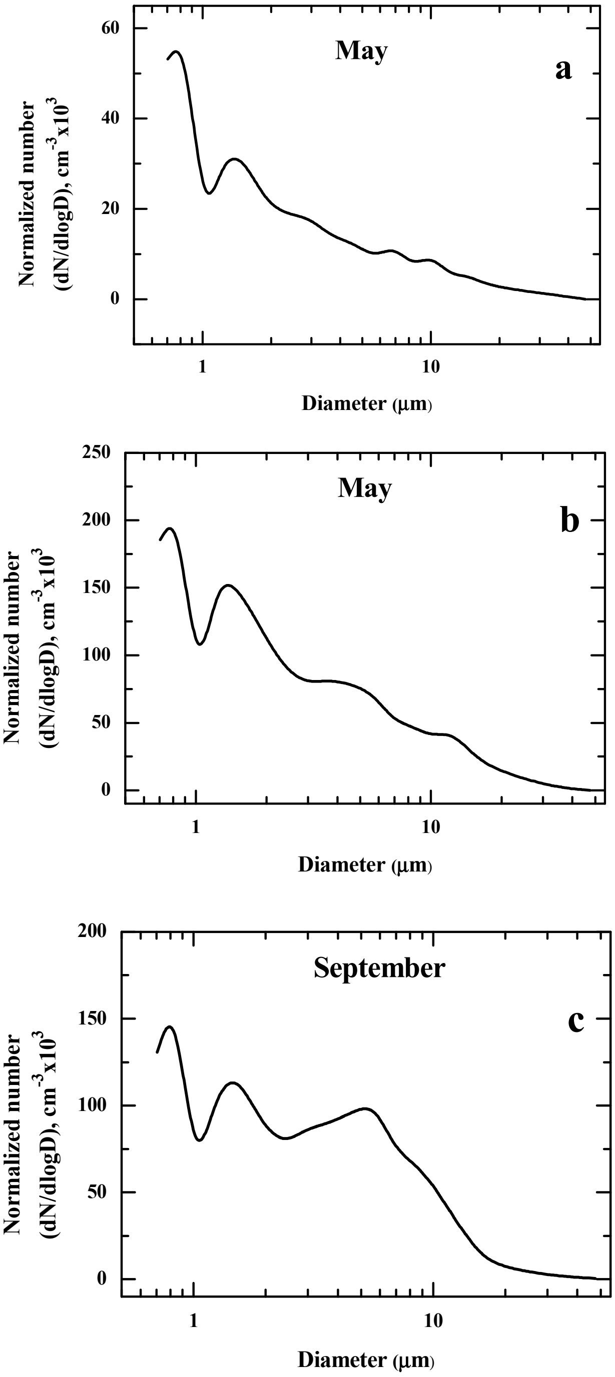

Figure S8. Normalized particle number size distributions of bioaerosol particles in the HV filter samples collected in May (a: # NHVA2019-0501-G-Q; b: # NHVA2019-0523-J-Q) and September (c, # NHVA2019-0923-J-Q) in 2019.

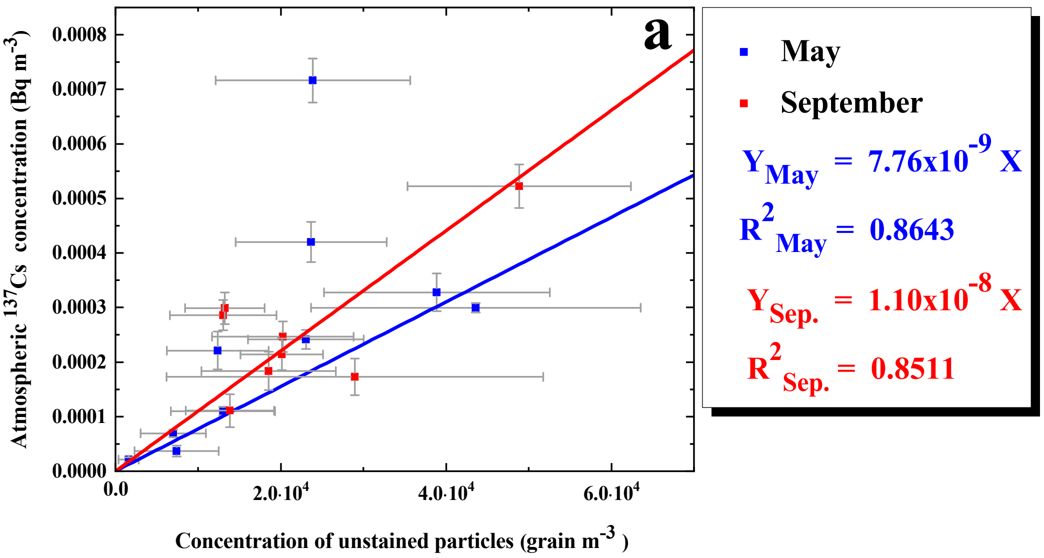

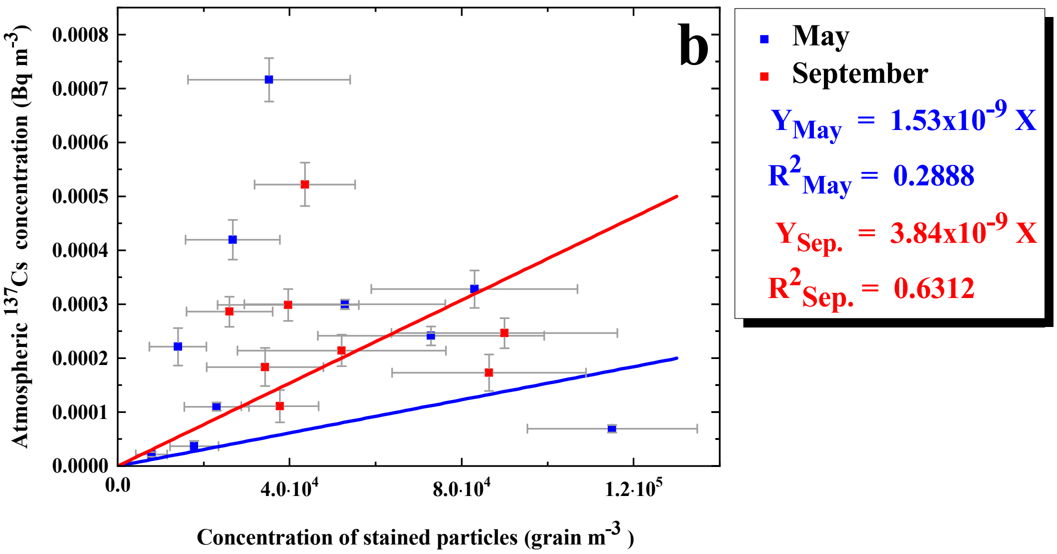

Figure S9. The concentration variations in atmospheric ^{137}Cs with the concentration of aerosol particles, respectively estimated from the HV aerosol filter samples collected in May 2019 (there were ten samples, n=10, highlighted in blue points) and in September 2019 (there were eight samples, n=8, highlighted in red points).

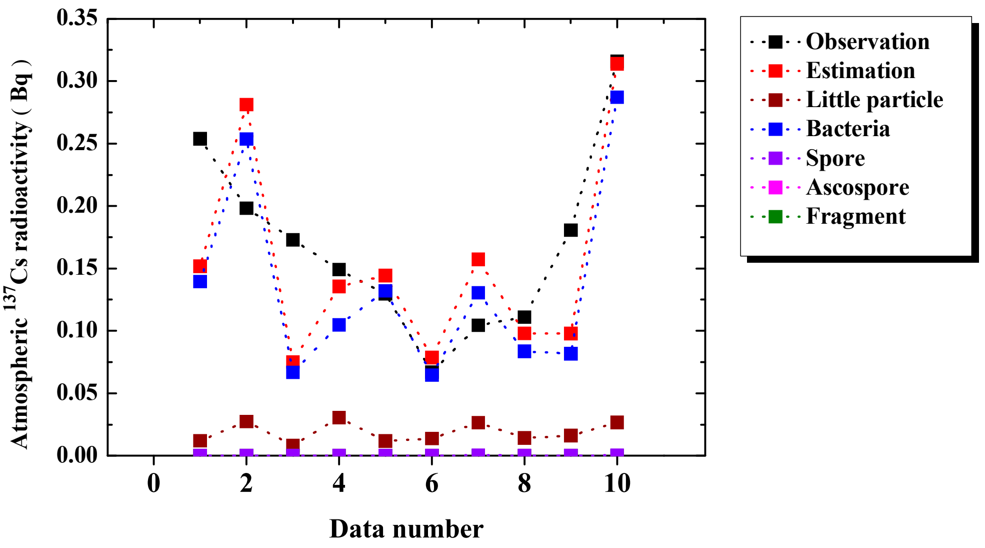

Figure S10. Bioaerosol particles' radioactivity contribution to ^{137}Cs radioactivity based on multiple linear regression Equation (2). The black squares are the measured values (*I*) of ^{137}Cs radioactivity in each HV aerosol filter sample. The red squares represent the estimated values (*I*) from Equation (2) in each sample. The rest of the symbols represent the contributed radioactivity of different bioaerosol particles to the radioactivity of ^{137}Cs based on statistical predictions from the multiple linear regression equation; detailed information is summarized in Table S5.

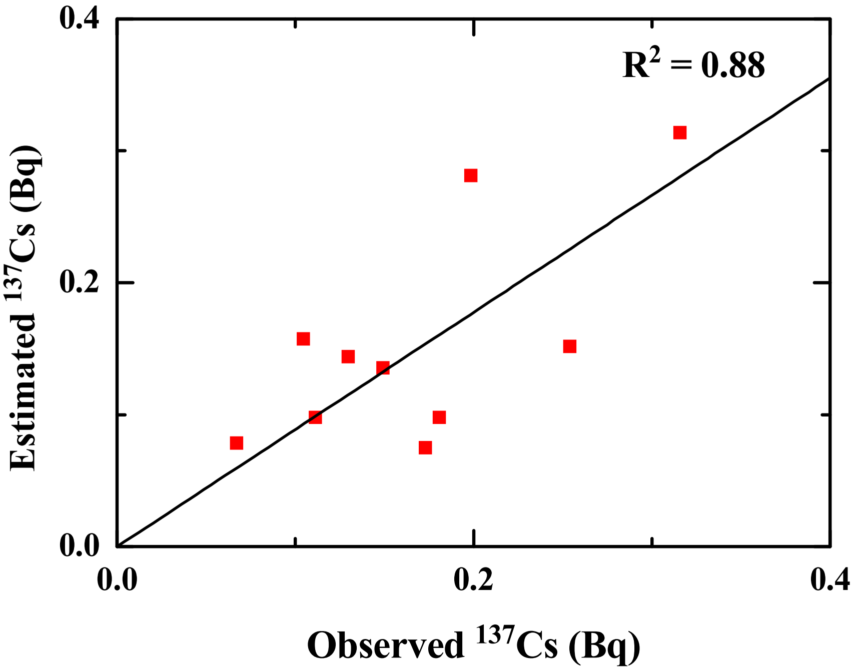

Figure S11. Correlation between observed values and estimated values for ^{137}Cs radioactivity.

Table S1. Radionuclides released from FDNPP accident modified from report of Ohara et al. (2011).

**Radionuclide**	**Half-Life**	**Released Amount (Bq)**	
		Japan Atomic Energy Agency (JAEA) 2011/5/12	Nuclear and Industrial Safety Agency (NISA) 2011/4/12
^{131}I	8.040 ± 0.001 days	1.5×10^{17}	1.6×10^{17}
^{132}I	2.30 ± 0.03 hours	—	4.7×10^{14}
^{133}I	20.8 ± 0.2 hours	—	6.8×10^{14}
^{134}Cs	2.062 ± 0.005 years	—	1.8×10^{16}
^{137}Cs	30.17 ± 0.05 years	1.3×10^{16}	1.5×10^{16}
^{106}Ru	368.0 ± 1.6 days	—	2.1×10^{9}
^{129m}Te	33.61 days	—	3.3×10^{15}
^{132}Te	78.2 ± 0.8 hours	—	7.6×10^{14}
^{144}Ce	284.4 ± 0.3 days	—	1.1×10^{13}
^{133}Xe	5.25 ± 0.02 days	—	1.1×10^{19}

Table S2. The information of samples in May and September 2019, giving the name and the sampling time.

Sample name	Starting time		Ending time	
	Year/Month/Day	a.m./p.m.	Year/Month/Day	a.m./p.m.
NHVA-20190501-D-Q	2019/4/26	6:00 p.m.	2019/4/27	6:00 a.m.
NHVA-20190501-L-Q	2019/4/30	6:00 p.m.	2019/5/1	6:00 a.m.
NHVA-20190511-G-Q	2019/5/7	6:00 p.m.	2019/5/8	6:00 a.m.
NHVA-20190511-J-Q	2019/5/10	6:00 p.m.	2019/5/11	6:00 a.m.
NHVA-20190523-A-Q	2019/5/11	6:00 p.m.	2019/5/12	6:00 a.m.
NHVA-20190523-J-Q	2019/5/15	6:00 p.m.	2019/5/16	6:00 a.m.
NHVA-20190923-H-Q	2019/9/20	6:00 p.m.	2019/9/21	6:00 a.m.
NHVA-20190923-J-Q	2019/9/21	6:00 p.m.	2019/9/22	6:00 a.m.
NHVA-20190923-L-Q	2019/9/22	6:00 p.m.	2019/9/23	6:00 a.m.

NHVA-20190929-B-Q	2019/9/23	6:00 p.m.	2019/9/24	6:00 a.m.
NHVA-20190929-H-Q	2019/9/26	6:00 p.m.	2019/9/27	6:00 a.m.
NHVA-20190501-G-Q	2019/4/28	6:00 a.m.	2019/4/28	6:00 p.m.
NHVA-20190511-L-Q	2019/5/10	6:00 a.m.	2019/5/10	6:00 p.m.
NHVA-20190523-K-Q	2019/5/12	6:00 a.m.	2019/5/12	6:00 p.m.
NHVA-20190523-I-Q	2019/5/15	6:00 a.m.	2019/5/15	6:00 p.m.
NHVA-20190923-G-Q	2019/9/20	6:00 a.m.	2019/9/20	6:00 p.m.
NHVA-20190923-K-Q	2019/9/22	6:00 a.m.	2019/9/22	6:00 p.m.

Table S3. All sampling information of the aerosol filters.

No.	Sample ID	Sampling Year	Sampling Volumes (m^3)	^{137}Cs in the Filter (Bq/m^3)	Error	Concentration of ^{137}Cs in the Filter (Bq/m^3)	Error
1	NHVA-20190124-Sat-Q	2019	2538.82	0.1091	0.0196	4.3E-05	7.7E-06
2	NHVA-20190124-Sun-Q	2019	2539.15	0.0937	0.0191	3.7E-05	7.5E-06
3	NHVA-20190124-Mon-Q	2019	2538.56	0.0946	0.0192	3.7E-05	7.6E-06
4	NHVA-20190124-Tue-Q	2019	2538.56	0.1293	0.0211	5.1E-05	8.3E-06
5	NHVA-20190124-Wed-Q	2019	2538.56	0.0901	0.0200	3.6E-05	7.9E-06
6	NHVA-20190124-Thr-Q	2019	2538.23	0.1311	0.0200	5.2E-05	7.9E-06
7	NHVA-20190124-Fri-Q	2019	2116.46	0.4292	0.0265	2.0E-04	1.3E-05
8	NHVA-20190124-Imp-Q	2019	2116.46	0.2604	0.0225	1.2E-04	1.1E-05
9	NHVA-20190124-Ura-Q	2019	2116.46	0.3228	0.0251	1.5E-04	1.2E-05
10	NHVA-20190124-Nep-Q	2019	1867.40	0.1729	0.0206	9.3E-05	1.1E-05
11	NHVA-20190124-QB(D)	2019	1867.40	0.0620	0.0200	3.3E-05	1.1E-05
12	NHVF-20190124-Q	2019	25728.81	0.5803	0.0274	2.3E-05	1.1E-06
13	NHVR-20190124-Q	2019	928.00	0.0816	0.0103	8.8E-05	1.1E-05
14	TJHV-20190124-Q	2019	18549.04	0.4945	0.0169	2.7E-05	9.1E-07
15	NHVA-20190301-Sat-Q	2019	5926.96	0.3034	0.0235	5.1E-05	4.0E-06
16	NHVA-20190301-Sun-Q	2019	5926.96	0.2588	0.0234	4.4E-05	3.9E-06
17	NHVA-20190301-Mon-Q	2019	5926.96	0.2242	0.0230	3.8E-05	3.9E-06
18	NHVA-20190301-Tue-Q	2019	5926.96	0.1839	0.0213	3.1E-05	3.6E-06
19	NHVA-20190301-Thr-Q	2019	6720.42	0.2142	0.0217	3.2E-05	3.2E-06
20	NHVA-20190301-QB(D)	2019	6720.42	0.0621	0.0205	9.2E-06	3.1E-06

No.	Sample ID	Sampling Year	Sampling Volumes (m^3)	^{137}Cs in the Filter (Bq/m^3)	Error	Concentration of ^{137}Cs in the Filter (Bq/m^3)	Error
21	NHVF-20190301-Q	2019	32059.88	0.7306	0.0284	2.3E-05	8.9E-07
22	NHVR-20190301-Q	2019	1993.92	0.1150	0.0125	5.8E-05	6.3E-06
23	TJHV-20190301-Q	2019	9674.72	0.5099	0.0148	5.3E-05	1.5E-06
24	TJHV-20190315-Q	2019	9525.10	0.3607	0.0136	3.8E-05	1.4E-06
25	NHVA-20190320-Wed-Q	2019	604.72	0.0391	0.0153	6.5E-05	2.5E-05
26	NHVA-20190320-Thr-Q	2019	604.72	0.0890	0.0163	1.5E-04	2.7E-05
27	NHVA-20190320-Fri-Q	2019	604.72	0.0594	0.0162	9.8E-05	2.7E-05
28	NHVA-20190320-Imp-Q	2019	604.72	0.0690	0.0163	1.1E-04	2.7E-05
29	NHVA-20190320-Ura-Q	2019	604.72	0.0782	0.0169	1.3E-04	2.8E-05
30	NHVA-20190320-Nep-Q	2019	604.72	0.0284	0.0157	4.7E-05	2.6E-05
31	NHVA-20190320-0-Q	2019	604.63	0.0426	0.0049	7.0E-05	8.1E-06
32	NHVA-20190320-0-Q	2019	604.63	0.0946	0.0064	1.6E-04	1.1E-05
33	NHVA-20190327-Sat-Q	2019	5926.96	0.3610	0.0100	6.1E-05	1.7E-06
34	NHVA-20190327-Sun-Q	2019	5926.87	0.7431	0.0172	1.3E-04	2.9E-06
35	NHVA-20190327-Mon-Q	2019	5926.96	0.6527	0.0175	1.1E-04	3.0E-06
36	NHVA-20190327-Tue-Q	2019	4152.88	0.3467	0.0158	8.3E-05	3.8E-06
37	NHVA-20190327-QB(D)	2019	4153.88	0.1568	0.0126	3.8E-05	3.0E-06
38	NHVF-20190327-Q	2019	22579.74	1.6459	0.0366	7.3E-05	1.6E-06
39	NHVR-20190327-Q	2019	2152.80	0.2854	0.0130	1.3E-04	6.0E-06
40	NHVA-20190425-Sat-Q	2019	3126.27	0.2783	0.0129	8.9E-05	4.1E-06
41	NHVA-20190425-Sun-Q	2019	3127.27	0.3278	0.0100	1.0E-04	3.2E-06
42	NHVA-20190425-Mon-Q	2019	3128.27	0.3564	0.0149	1.1E-04	4.8E-06

No.	Sample ID	Sampling Year	Sampling Volumes (m^3)	^{137}Cs in the Filter (Bq/m^3)	Error	Concentration of ^{137}Cs in the Filter (Bq/m^3)	Error
43	NHVA-20190425-Tue-Q	2019	3129.27	0.6669	0.0181	2.1E-04	5.8E-06
44	NHVA-20190425-QB(D)	2019	3130.27	0.1000	0.0101	3.2E-05	3.2E-06
45	NHVR-20190425-Q	2019	3131.27	0.1458	0.0110	4.7E-05	3.5E-06
46	NHVA-20190501-Sun-Q	2019	604.72	0.0185	0.0040	3.1E-05	6.6E-06
47	NHVA-20190501-Tue-Q	2019	604.72	0.0130	0.0026	2.2E-05	4.4E-06
48	NHVA-20190501-Thr-Q	2019	604.72	0.0370	0.0052	6.1E-05	8.7E-06
49	NHVA-20190501-0-Q	2019	604.63	0.0146	0.0036	2.4E-05	6.0E-06
50	NHVA-20190501-Wed-Q	2019	604.72	0.0222	0.0060	3.7E-05	9.9E-06
51	NHVA-20190501-Fri-Q	2019	604.72	0.0210	0.0055	3.5E-05	9.1E-06
52	NHVA-20190501-Imp-Q	2019	604.72	0.0239	0.0042	4.0E-05	6.9E-06
53	NHVA-20190501-Ura-Q	2019	604.72	0.0205	0.0053	3.4E-05	8.8E-06
54	NHVA-20190501-0-Q	2019	604.63	0.0419	0.0042	6.9E-05	7.0E-06
55	NHVA-20190506-Sat-Q	2019	5926.87	0.1953	0.0116	3.3E-05	2.0E-06
56	NHVA-20190511-Sat-Q	2019	604.72	0.0967	0.0081	1.6E-04	1.3E-05
57	NHVA-20190511-Thr-Q	2019	604.72	0.0720	0.0090	1.2E-04	1.5E-05
58	NHVA-20190511-0-Q	2019	604.63	0.1041	0.0075	1.7E-04	1.2E-05
59	NHVA-20190511-Wed-Q	2019	604.72	0.0665	0.0048	1.1E-04	7.9E-06
60	NHVA-20190511-Fri-Q	2019	604.72	0.1038	0.0088	1.7E-04	1.5E-05
61	NHVA-20190511-Imp-Q	2019	604.72	0.0832	0.0059	1.4E-04	9.8E-06
62	NHVA-20190511-Nep-Q	2019	604.72	0.1460	0.0106	2.4E-04	1.8E-05
63	NHVA-20190511-Ura-Q	2019	604.72	0.1498	0.0055	2.5E-04	9.0E-06
64	NHVA-20190511-0-Q	2019	604.63	0.1811	0.0053	3.0E-04	8.8E-06

No.	Sample ID	Sampling Year	Sampling Volumes (m^3)	^{137}Cs in the Filter (Bq/m^3)	Error	Concentration of ^{137}Cs in the Filter (Bq/m^3)	Error
65	NHVA-20190523-Sat-Q	2019	604.72	0.1337	0.0209	2.2E-04	3.5E-05
66	NHVA-20190523-Mon-Q	2019	5926.96	0.5948	0.0159	1.0E-04	2.7E-06
67	NHVA-20190523-Sun-Q	2019	5926.96	1.5653	0.0228	2.6E-04	3.9E-06
68	NHVA-20190523-Tue-Q	2019	5926.96	1.3817	0.0219	2.3E-04	3.7E-06
69	NHVA-20190523-Thr-Q	2019	604.72	0.1634	0.0212	2.7E-04	3.5E-05
70	NHVA-20190523-0-Q	2019	604.63	0.1650	0.0167	2.7E-04	2.8E-05
71	NHVA-20190523-Wed-Q	2019	604.72	0.2342	0.0223	3.9E-04	3.7E-05
72	NHVA-20190523-Fri-Q	2019	604.72	0.1520	0.0206	2.5E-04	3.4E-05
73	NHVA-20190523-Imp-Q	2019	604.72	0.2538	0.0222	4.2E-04	3.7E-05
74	NHVA-20190523-Nep-Q	2019	604.72	0.1983	0.0211	3.3E-04	3.5E-05
75	NHVA-20190523-Ura-Q	2019	604.72	0.4331	0.0244	7.2E-04	4.0E-05
76	NHVA-20190523-0-Q	2019	604.63	0.1620	0.0211	2.7E-04	3.5E-05
77	NHVA-20190523-QB(D)	2019	605.63	0.1670	0.0134	2.8E-04	2.2E-05
78	NHVA-20190523-QB(D)	2019	606.63	0.1224	0.0167	2.0E-04	2.8E-05
79	NHVA-20190620-Sat-Q	2019	5926.96	1.6333	0.0233	2.8E-04	3.9E-06
80	NHVA-20190620-Mon-Q	2019	5926.96	0.6349	0.0100	1.1E-04	1.7E-06
81	NHVA-20190620-Sun-Q	2019	5926.96	0.5207	0.0106	8.8E-05	1.8E-06
82	NHVA-20190620-Tue-Q	2019	5926.96	1.0112	0.0158	1.7E-04	2.7E-06
83	NHVA-20190620-QB(D)	2019	5927.96	0.1000	0.0121	1.7E-05	2.0E-06
84	NHVR-20190620-Q	2019	3968.92	0.3513	0.0136	8.9E-05	3.4E-06
85	NHVA-20190718-Sat-Q	2019	5926.96	0.7295	0.0109	1.2E-04	1.8E-06
86	NHVA-20190718-Mon-Q	2019	5926.96	0.8763	0.0114	1.5E-04	1.9E-06

No.	Sample ID	Sampling Year	Sampling Volumes (m³)	^{137}Cs in the Filter (Bq/m³)	Error	Concentration of ^{137}Cs in the Filter (Bq/m³)	Error
87	NHVA-20190718-Sun-Q	2019	5926.87	0.5195	0.0166	8.8E-05	2.8E-06
88	NHVA-20190718-Tue-Q	2019	5926.96	1.4316	0.0236	2.4E-04	4.0E-06
89	NHVA-20190718-QB(D)	2019	5927.96	0.0265	0.0076	4.5E-06	1.3E-06
90	NHVR-20190718-Q	2019	6526.80	0.5555	0.0070	8.5E-05	1.1E-06
91	NHVA-20190822-Sat-Q	2019	5926.87	1.5565	0.0229	2.6E-04	3.9E-06
92	NHVA-20190822-Mon-Q	2019	5926.96	1.1899	0.0220	2.0E-04	3.7E-06
93	NHVA-20190822-Tue-Q	2019	5926.96	1.0224	0.0182	1.7E-04	3.1E-06
94	NHVA-20190822-Thr-Q	2019	5926.96	0.8019	0.0169	1.4E-04	2.8E-06
95	NHVR-20190822-Q	2019	3177.97	0.7013	0.0131	2.2E-04	4.1E-06
96	NHVA-20190919-Sat-Q	2019	5926.96	0.9051	0.0203	1.5E-04	3.4E-06
97	NHVA-20190919-Mon-Q	2019	5926.96	1.4358	0.0222	2.4E-04	3.7E-06
98	NHVA-20190919-Sun-Q	2019	5926.87	1.4690	0.0243	2.5E-04	4.1E-06
99	NHVA-20190919-Tue-Q	2019	5820.95	1.2674	0.0174	2.2E-04	3.0E-06
100	NHVA-20190919-Thr-Q	2019	5821.95	0.1961	0.0085	3.4E-05	1.5E-06
101	NHVA-20190919-0-Q	2019	5822.95	0.1259	0.0112	2.2E-05	1.9E-06
102	NHVA-20190923-Wed-Q	2019	604.72	0.1730	0.0170	2.9E-04	2.8E-05
103	NHVA-20190923-Fri-Q	2019	604.72	0.1490	0.0169	2.5E-04	2.8E-05
104	NHVA-20190923-Imp-Q	2019	604.72	0.1098	0.0200	1.8E-04	3.3E-05
105	NHVA-20190923-Nep-Q	2019	604.72	0.1296	0.0176	2.1E-04	2.9E-05
106	NHVA-20190923-Ura-Q	2019	604.72	0.0671	0.0183	1.1E-04	3.0E-05
107	NHVA-20190923-0-Q	2019	604.63	0.1045	0.0204	1.7E-04	3.4E-05
108	NHVA-20190923-QB(D)	2019	605.63	0.0304	0.0175	5.0E-05	2.9E-05

No.	Sample ID	Sampling Year	Sampling Volumes (m^3)	^{137}Cs in the Filter (Bq/m^3)	Error	Concentration of ^{137}Cs in the Filter (Bq/m^3)	Error
109	NHVR-20190923-Q	2019	639.66	0.0899	0.0200	1.4E-04	3.1E-05
110	NHVA-20190929-Sat-Q	2019	604.72	0.1455	0.0202	2.4E-04	3.3E-05
111	NHVA-20190929-Mon-Q	2019	604.72	0.1110	0.0214	1.8E-04	3.5E-05
112	NHVA-20190929-Sun-Q	2019	604.72	0.1847	0.0213	3.1E-04	3.5E-05
113	NHVA-20190929-Tue-Q	2019	604.72	0.1762	0.0205	2.9E-04	3.4E-05
114	NHVA-20190929-Thr-Q	2019	604.72	0.1224	0.0167	2.0E-04	2.8E-05
115	NHVA-20190929-0-Q	2019	604.63	0.2218	0.0218	3.7E-04	3.6E-05
116	NHVA-20190929-Fri-Q	2019	604.72	0.1806	0.0176	3.0E-04	2.9E-05
117	NHVA-20190929-Imp-Q	2019	604.72	0.2266	0.0222	3.7E-04	3.7E-05
118	NHVA-20190929-Nep-Q	2019	604.72	0.1963	0.0213	3.2E-04	3.5E-05
119	NHVA-20190929-Ura-Q	2019	604.72	0.3158	0.0242	5.2E-04	4.0E-05
120	NHVA-20190929-0-Q	2019	604.63	0.1662	0.0234	2.7E-04	3.9E-05
121	NHVA-20190929-QB(D)	2019	605.63	0.0417	0.0189	6.9E-05	3.1E-05

Table S4. Several instrumental specifications of meteorological monitoring modified from Appendix of Ishizuka et al. (2017).

Monitoring Object	Instrument	Manufacture	Model
**Precipitation**	Tipping bucket rain gauge	Takeda Keiki Kougyou	TKF-1
**Humidity (RH)**	Capacitive chip	Vaisala Corp.	HMP155D
**Wind speed**	Three cups anemometer	R. M. Young	3102
**Gust wind speed**	Sonic anemometer	R. M. Young	81000
**Air temperature**	Pt resistance thermometer	Vaisala Corp.	HMP155D
**Moisture**	ADR sensor	Delta-T Devices Ltd.	Theta probe ML2x

Table S5. Classification of bioaerosol particles based on different sizes. AR (aspect ratio) represents the ratio of the major to the minor. The major and the minor were the primary and secondary axes of the best-fitting ellipse. Circularity is defined as $4\pi\times$(area) / (squared circumference). A value of circularity of 1 represents the particle that can be regarded as a perfect circle. If the value approaches 0, it indicates that the shape of the particle is elongated. When the AR is greater than 4 and the circularity is less than 0.45, the particle is defined as a fragment.

Species	Parameter		Size		References	Appearance Examples
	AR	Circularity	Major	Minor		
Fragment	>4	<0.45				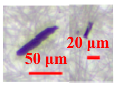
Little particle	<4	>0.45	0.65-1.0µm	0.65-1.0µm		
Bacteria	<4	>0.45	1.0-1.8µm	1.0-1.8µm	Hoorman et al. (2011)	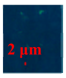
Basidiospore	<4	>0.45	1.8-10µm	1.8-5µm	Yamamoto et al. (2012)	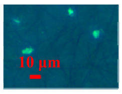
Ascospore	<4	>0.45	10-60µm	5-15µm		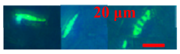
Pollen	<4	>0.7		>15µm	Stanley and Linskins (1974); Kelly et al. (2002)	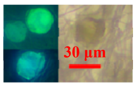
Others						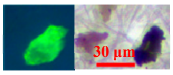

Table S6. Each coefficient in Equation (2). a_i is the coefficient of each bioaerosol, referring to the ^{137}Cs radioactivity accumulated in each type of bioaerosol within each HV aerosol filter sample. The statistical analysis was made by regression analysis for 10 samples. Asterisk (*) indicates a significant level.

a_i	Coefficient Value (Bq μm^{-2})	b_0	Residual Radioactivity (Bq)
a_1	1.11×10^{-8}	b_0	0.19
a_2	3.08×10^{-8} *		
a_3	9.14×10^{-12}		
a_4	0.00		
a_5	0.00		
a_6	0.00		

Table S7. Contribution values of each species of bioaerosols to ^{137}Cs radioactivity. There were ten data (eight in September, and two on 15 May; consideration of bioaerosols as the main carriers). The observed values (Bq) of ^{137}Cs radioactivity are obtained from the HV aerosol filter samples (183.2 mm × 234 mm). The estimated values (Bq) are based on a prediction of Equation (2) in each sample. The predicted radioactivity (Bq) in little particles, bacteria, spores, ascospores, and fragments (without considering pollens because of seasonality) was calculated from statistical analysis in multiple linear regression Equation (2). The residual values were involved in the difference between the measured value and the estimated value.

Data Number	Measured Value (Bq)	Estimated Value (Bq)	Little Particle (Bq)	Bacteria (Bq)	Spore (Bq)	Ascospore (Bq)	Fragment (Bq)	Residual Value (Bq)
1	0.2538	0.1516	0.0119	0.1395	0.0002	0.0000	0.0000	0.1023
2	0.1983	0.2812	0.0275	0.2536	0.0002	0.0000	0.0000	-0.0829
3	0.1730	0.0751	0.0083	0.0667	0.0001	0.0000	0.0000	0.0980
4	0.1490	0.1355	0.0306	0.1048	0.0002	0.0000	0.0000	0.0134
5	0.1296	0.1442	0.0118	0.1321	0.0003	0.0000	0.0000	-0.0146
6	0.0671	0.0786	0.0139	0.0645	0.0001	0.0000	0.0000	-0.0114
7	0.1045	0.1573	0.0264	0.1305	0.0004	0.0000	0.0000	-0.0528
8	0.1110	0.0980	0.0143	0.0835	0.0002	0.0000	0.0000	0.0130
9	0.1806	0.0978	0.0161	0.0817	0.0001	0.0000	0.0000	0.0828
10	0.3158	0.3139	0.0266	0.2870	0.0004	0.0000	0.0000	0.0019

Table S8. The variation in atmospheric ^{137}Cs concentration, precipitation, temperature, relative humidity, wind speed, and gust collected at sampling location.

Sampling Date	Concentration of ^{137}Cs (Bq/m^3)	Precipitation (mm)	Temperature (°C)	Relative Humidity, RH (%)	Wind Speed (m/s)	Gust (m/s)
4/26	2.15E-05	15	4.9	97.8	0.14	2.01
4/28	3.68E-05	0	8.1	62.7	0.45	3.52
4/30	6.92E-05	10	11.7	91.7	0.21	2.52
5/7	1.10E-04	1.4	9.1	66.9	0.5	3.52
5/10	2.70E-04	0	14	52.5	0.32	3.52
5/11	2.21E-04	0	14.1	71.7	0.22	2.52
5/12	7.16E-04	0	9.3	78.5	0.27	4.53
5/15	3.74E-04	4.8	13.9	90.3	0.09	4.03
9/20	2.66E-04	0	14.9	87	0.02	3.02
9/21	2.14E-04	0	15.9	92.3	0.02	2.52
9/22	1.42E-04	1	17	97.6	0.01	3.02
9/23	1.84E-04	3.81	21.5	92.6	0.14	6.04
9/26	2.99E-04	0	13.9	86.8	0.09	4.03
9/28	5.22E-04	0	19.3	89.5	0.01	3.02
